# Supplementary material for: Mass mortality event of the giant barrel sponge Xestospongia sp.: population dynamics and size distribution in Koh Phangan, Gulf of Thailand
Source: PeerJ. 2023 Dec 12;11:e16561. doi: 10.7717/peerj.16561 (PMC10722979; doi:10.7717/peerj.16561)
Supplement: Supplemental Information 1 — Shown are the mean, the variance and the P-Value calculated for all study sites and survey times. The abundances of bleached Xestospongia individuals are concluded in the results of April 2015. The asterisk (*) show significant differences with p < 0.05. [file peerj-11-16561-s001.pdf]

		Mean		Variance		P-Value
		4m depth	6m depth	4m depth	6m depth	
Mae Haad	2015.1	-	-	-	-	-
	2015.2 bleached	10.7	2.3	142.3	6.3	0.179
	2016	-	-	-	-	-
Haad Khom	2015.1	10.3	7.7	20.3	3.5	0.111
	2015.2 bleached	11.3	5.7	139.9	10.3	0.150
	2016	2.0	1.5	1.2	1.1	0.219
Koh Yippon	2015.1	51.7	36.0	25.3	25.0	0.009 *
	2015.2 bleached	-	-	-	-	-
	2016	16.0	44.0	31.0	288.0	0.133
Hin Yippon	2015.1	29.3	31.3	86.3	82.3	0.401
	2015.2 bleached	-	-	-	-	-
	2016	24.7	33.0	252.3	392.0	0.334
