# Supplementary figures and images for: Mass mortality event of the giant barrel sponge Xestospongia sp.: population dynamics and size distribution in Koh Phangan, Gulf of Thailand
Source: PeerJ. 2023 Dec 12;11:e16561. doi: 10.7717/peerj.16561 (PMC10722979; doi:10.7717/peerj.16561)

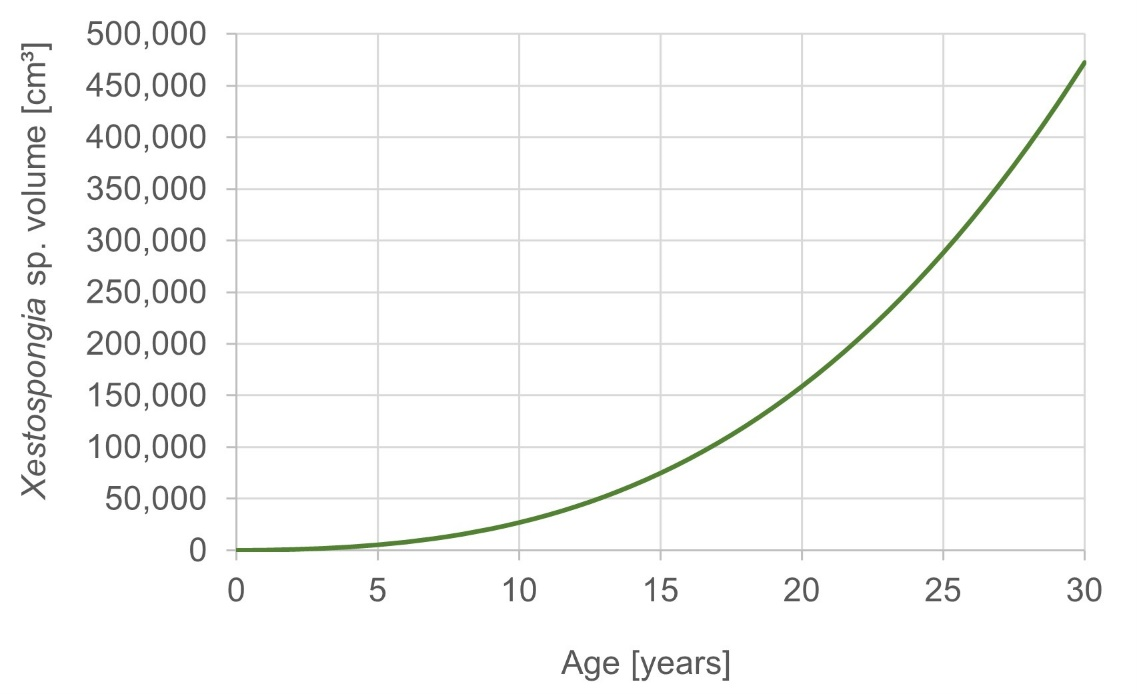

Supplement: Supplemental Information 2 — The y-axis shows the sponge volume, and the x-axis shows the sponge age of Xestospongia sp. based on the averaged model from McGrath et al., 2018. [file peerj-11-16561-s002.png]
